# 8-(4-Chloro­benzyl­idene)-4-(4-chloro­phen­yl)-2-phenyl-5,6,7,8-tetra­hydro­quinoline

**DOI:** 10.1107/S1600536810017769

**Published:** 2010-05-22

**Authors:** Fangfang Jian, Xin Zhai

**Affiliations:** a New Materials & Function Coordination Chemistry Laboratory, Qingdao University of Science & Technology, Qingdao 266042, People’s Republic of China

## Abstract

In the crystal structure of the title compound, C_28_H_21_Cl_2_N, π–π inter­actions link pairs of mol­ecules into centrosymmetric dimers with a distance of 3.756 (3) Å between the centroids of the pyridine rings. Weak inter­molecular C—H⋯Cl hydrogen bonds further link these dimers into chains propagating along [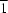
01]. The pyridine ring forms dihedral angles of 21.52 (1) and 55.87 (2)°, respectively, with the phenyl ring and the 4-chlorophenyl ring.

## Related literature

For applications of pyridyl-containing compounds, see: Yan *et al.* (2007 ▶); Barton & Ollis (1979 ▶); Katritzky & Marson (1984 ▶); Constable *et al.* (1994 ▶); Eryazici *et al.* (2006 ▶).
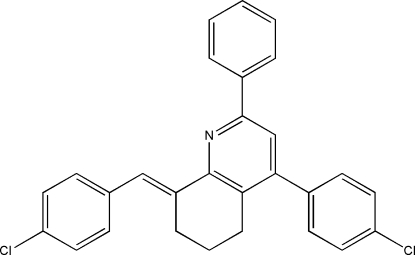


## Experimental

### 

#### Crystal data

C_28_H_21_Cl_2_N
*M* *_r_* = 442.36
Triclinic, 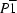

*a* = 10.0583 (10) Å
*b* = 10.6483 (10) Å
*c* = 10.8792 (10) Å
α = 82.028 (2)°
β = 89.345 (1)°
γ = 71.335 (2)°
*V* = 1092.53 (18) Å^3^
*Z* = 2
Mo *K*α radiation
μ = 0.31 mm^−1^
*T* = 295 K
0.23 × 0.20 × 0.19 mm

#### Data collection

Enraf–Nonius CAD-4 diffractometer
5765 measured reflections
3810 independent reflections
3211 reflections with *I* > 2σ(*I*)
*R*_int_ = 0.014
3 standard reflections every 100 reflections  intensity decay: none

#### Refinement

*R*[*F* ^2^ > 2σ(*F* ^2^)] = 0.036
*wR*(*F* ^2^) = 0.102
*S* = 1.06
3810 reflections
280 parameters
H-atom parameters constrained
Δρ_max_ = 0.20 e Å^−3^
Δρ_min_ = −0.30 e Å^−3^

### 

Data collection: *CAD-4 Software* (Enraf–Nonius, 1989 ▶); cell refinement: *CAD-4 Software* data reduction: *NRCVAX* (Gabe *et al.*, 1989 ▶); program(s) used to solve structure: *SHELXS97* (Sheldrick, 2008 ▶); program(s) used to refine structure: *SHELXL97* (Sheldrick, 2008 ▶); molecular graphics: *SHELXTL* (Sheldrick, 2008 ▶); software used to prepare material for publication: *WinGX* (Farrugia, 1999 ▶).

## Supplementary Material

Crystal structure: contains datablocks global, I. DOI: 10.1107/S1600536810017769/cv2704sup1.cif

Structure factors: contains datablocks I. DOI: 10.1107/S1600536810017769/cv2704Isup2.hkl

Additional supplementary materials:  crystallographic information; 3D view; checkCIF report

## Figures and Tables

**Table 1:** Hydrogen-bond geometry (Å, °)

*D*—H⋯*A*	*D*—H	H⋯*A*	*D*⋯*A*	*D*—H⋯*A*
C20—H20*A*⋯Cl1^i^	0.93	2.80	3.476 (2)	130

Symmetry code: (i) 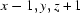.

**Fig. 1.**
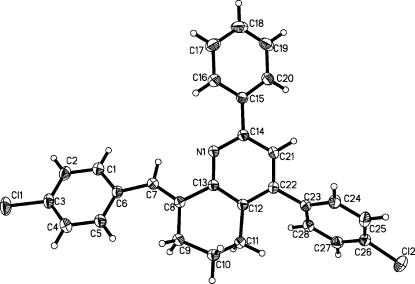
The structure of the title compound showing 30% probability displacement ellipsoids and the atom-numbering scheme.

## supplementary crystallographic information

### Comment

The pyridyl heterocyclic core occurs in many natural products (Barton & Ollis, 1979; Katritzky & Marson, 1984). It also plays an important role in various coordinating ligands (Constable *et al.*, 1994; Eryazici *et al.*, 2006)˙ Recently, the structure of 5,6,7,8-tetrahydroquinoline derivative has been reported (Yan *et al.*, 2007). Herein, we report the crystal structure of the title compound.

In (I) (Fig. 1), the bond lengths and angles are in a good agreement with those reported previously (Yan *et al.*, 2007). Rings N1/C13/C12/C22/C21/C14 (p1), C15-C20 (p2), C23-C28 (p3) and C1-C6 (p4) form the following dihedral angles - p1/p2 21.52 (1)°, p1/p3 55.87 (2)°, p1/p4 33.74 (1)°, p2/p3  67.85 (1)°, p2/p4 44.53 (2)° and p3/p4 81.57 (1)°.

The crystal packing is stabilized by hydrogen bonds and π-π interactions. π-π interaction link two molecules into centrosymmetric dimer with the distance of 3.756 (3) Å between the centroids of pyridine rings. Weak intermolecular C—H···Cl hydrogen bonds (Table 1) link further these dimers into chains propagated in direction [-101].

### Experimental

The title compound was synthesized by reaction of (*Z*)-2,6-dibenzylidenecyclohexanone (0.343 g, 1 mmol),ammonium acetate (3.0 g, 0.039 mol) and *N*-phenacylpyridinium bromide (0.280 g, 1.2 mmol) in refluxing methanol (15 ml) under stirring for 7 h. Single crystals suitable for x-ray measurements were obtained by recrystallization from ethanol at room temperature.

### Refinement

C-bound H atoms were positioned geometrically and allowed to ride on their parent atoms, with C—H = 0.93-0.97 Å and U_iso_(H)=1.2-1.5U_eq_(C).

### Crystal data

**Table:**

C_28_H_21_Cl_2_N	*Z* = 2
*M**_r_* = 442.36	*F*(000) = 460
Triclinic, *P*1	*D*_x_ = 1.345 Mg m^−^^3^
Hall symbol: -P 1	Melting point: 446 K
*a* = 10.0583 (10) Å	Mo *K*α radiation, λ = 0.71073 Å
*b* = 10.6483 (10) Å	Cell parameters from 25 reflections
*c* = 10.8792 (10) Å	θ = 4–14°
α = 82.028 (2)°	µ = 0.31 mm^−^^1^
β = 89.345 (1)°	*T* = 295 K
γ = 71.335 (2)°	Block, colorless
*V* = 1092.53 (18) Å^3^	0.23 × 0.20 × 0.19 mm

### Data collection

**Table:**

Enraf–Nonius CAD-4 diffractometer	*R*_int_ = 0.014
Radiation source: fine-focus sealed tube	θ_max_ = 25.0°, θ_min_ = 1.9°
graphite	*h* = −10→11
ω scans	*k* = −10→12
5765 measured reflections	*l* = −12→12
3810 independent reflections	3 standard reflections every 100 reflections
3211 reflections with *I* > 2σ(*I*)	intensity decay: none

### Refinement

**Table:**

Refinement on *F*^2^	Primary atom site location: structure-invariant direct methods
Least-squares matrix: full	Secondary atom site location: difference Fourier map
*R*[*F*^2^ > 2σ(*F*^2^)] = 0.036	Hydrogen site location: inferred from neighbouring sites
*wR*(*F*^2^) = 0.102	H-atom parameters constrained
*S* = 1.06	*w* = 1/[σ^2^(*F*_o_^2^) + (0.049*P*)^2^ + 0.2862*P*] where *P* = (*F*_o_^2^ + 2*F*_c_^2^)/3
3810 reflections	(Δ/σ)_max_ < 0.001
280 parameters	Δρ_max_ = 0.20 e Å^−^^3^
0 restraints	Δρ_min_ = −0.30 e Å^−^^3^

### Special details

**Table:**

Geometry. All esds (except the esd in the dihedral angle between two l.s. planes) are estimated using the full covariance matrix. The cell esds are taken into account individually in the estimation of esds in distances, angles and torsion angles; correlations between esds in cell parameters are only used when they are defined by crystal symmetry. An approximate (isotropic) treatment of cell esds is used for estimating esds involving l.s. planes.
Refinement. Refinement of *F*^2^ against ALL reflections. The weighted *R*-factor wR and goodness of fit *S* are based on *F*^2^, conventional *R*-factors *R* are based on *F*, with *F* set to zero for negative *F*^2^. The threshold expression of *F*^2^ > σ(*F*^2^) is used only for calculating *R*-factors(gt) etc. and is not relevant to the choice of reflections for refinement. *R*-factors based on *F*^2^ are statistically about twice as large as those based on *F*, and *R*- factors based on ALL data will be even larger.

### Fractional atomic coordinates and isotropic or equivalent isotropic displacement parameters (Å^2^)

**Table:**

	*x*	*y*	*z*	*U*_iso_*/*U*_eq_	
Cl1	0.76136 (6)	−0.12681 (6)	−0.49616 (5)	0.06911 (19)	
Cl2	−0.44155 (6)	0.60618 (5)	0.38242 (6)	0.0742 (2)	
N1	0.17509 (14)	−0.06763 (13)	0.09541 (12)	0.0368 (3)	
C1	0.45331 (19)	−0.14359 (18)	−0.27005 (16)	0.0451 (4)	
H1B	0.3983	−0.1989	−0.2522	0.054*	
C2	0.5506 (2)	−0.17024 (19)	−0.36113 (17)	0.0489 (4)	
H2A	0.5600	−0.2415	−0.4049	0.059*	
C3	0.63328 (18)	−0.09032 (19)	−0.38617 (16)	0.0458 (4)	
C4	0.61789 (19)	0.0167 (2)	−0.32375 (17)	0.0499 (5)	
H4A	0.6740	0.0710	−0.3420	0.060*	
C5	0.51923 (19)	0.04352 (18)	−0.23411 (16)	0.0451 (4)	
H5A	0.5089	0.1167	−0.1928	0.054*	
C6	0.43443 (17)	−0.03640 (16)	−0.20368 (15)	0.0374 (4)	
C7	0.33130 (17)	−0.02208 (16)	−0.10601 (15)	0.0379 (4)	
H7A	0.2986	−0.0948	−0.0881	0.045*	
C8	0.27524 (16)	0.07649 (16)	−0.03750 (15)	0.0350 (4)	
C9	0.30828 (19)	0.20661 (17)	−0.04971 (16)	0.0416 (4)	
H9A	0.2820	0.2535	−0.1333	0.050*	
H9B	0.4088	0.1863	−0.0380	0.050*	
C10	0.23390 (19)	0.29860 (17)	0.04218 (17)	0.0441 (4)	
H10A	0.2800	0.2652	0.1235	0.053*	
H10B	0.2405	0.3873	0.0166	0.053*	
C11	0.08054 (18)	0.30767 (16)	0.05054 (16)	0.0424 (4)	
H11A	0.0344	0.3694	0.1073	0.051*	
H11B	0.0336	0.3405	−0.0305	0.051*	
C12	0.07233 (17)	0.17042 (16)	0.09631 (14)	0.0355 (4)	
C13	0.17015 (16)	0.05872 (16)	0.05496 (14)	0.0345 (4)	
C14	0.08518 (17)	−0.08920 (16)	0.18078 (15)	0.0369 (4)	
C15	0.09818 (18)	−0.23138 (16)	0.22446 (15)	0.0386 (4)	
C16	0.2229 (2)	−0.33247 (17)	0.21094 (17)	0.0473 (4)	
H16A	0.2978	−0.3108	0.1735	0.057*	
C17	0.2370 (2)	−0.46471 (19)	0.25232 (19)	0.0578 (5)	
H17A	0.3211	−0.5313	0.2426	0.069*	
C18	0.1272 (3)	−0.4986 (2)	0.30786 (19)	0.0610 (6)	
H18A	0.1370	−0.5877	0.3361	0.073*	
C19	0.0030 (2)	−0.3998 (2)	0.32122 (18)	0.0578 (5)	
H19A	−0.0716	−0.4223	0.3585	0.069*	
C20	−0.0120 (2)	−0.26712 (18)	0.27962 (16)	0.0469 (4)	
H20A	−0.0968	−0.2011	0.2887	0.056*	
C21	−0.01285 (18)	0.01573 (17)	0.22810 (15)	0.0399 (4)	
H21A	−0.0728	−0.0021	0.2887	0.048*	
C22	−0.02151 (17)	0.14700 (16)	0.18517 (15)	0.0369 (4)	
C23	−0.12942 (17)	0.25829 (16)	0.23450 (15)	0.0380 (4)	
C24	−0.13747 (19)	0.26296 (18)	0.36124 (16)	0.0460 (4)	
H24A	−0.0763	0.1944	0.4158	0.055*	
C25	−0.2349 (2)	0.3679 (2)	0.40779 (18)	0.0513 (5)	
H25A	−0.2389	0.3706	0.4929	0.062*	
C26	−0.32574 (19)	0.46814 (18)	0.32692 (18)	0.0467 (4)	
C27	−0.32402 (19)	0.46397 (18)	0.20151 (17)	0.0472 (4)	
H27A	−0.3884	0.5307	0.1478	0.057*	
C28	−0.22516 (19)	0.35909 (18)	0.15609 (16)	0.0444 (4)	
H28A	−0.2230	0.3563	0.0710	0.053*	

### Atomic displacement parameters (Å^2^)

**Table:**

	*U*^11^	*U*^22^	*U*^33^	*U*^12^	*U*^13^	*U*^23^
Cl1	0.0586 (3)	0.0873 (4)	0.0505 (3)	−0.0087 (3)	0.0250 (2)	−0.0108 (3)
Cl2	0.0743 (4)	0.0533 (3)	0.0875 (4)	−0.0030 (3)	0.0244 (3)	−0.0288 (3)
N1	0.0408 (8)	0.0361 (7)	0.0351 (7)	−0.0139 (6)	0.0084 (6)	−0.0076 (6)
C1	0.0467 (10)	0.0434 (10)	0.0459 (10)	−0.0140 (8)	0.0093 (8)	−0.0106 (8)
C2	0.0527 (11)	0.0490 (11)	0.0418 (10)	−0.0086 (9)	0.0090 (8)	−0.0146 (8)
C3	0.0396 (9)	0.0550 (11)	0.0329 (9)	−0.0034 (8)	0.0076 (7)	−0.0024 (8)
C4	0.0466 (10)	0.0575 (11)	0.0476 (10)	−0.0201 (9)	0.0113 (8)	−0.0067 (9)
C5	0.0487 (10)	0.0481 (10)	0.0433 (10)	−0.0199 (8)	0.0123 (8)	−0.0129 (8)
C6	0.0365 (9)	0.0383 (9)	0.0341 (8)	−0.0079 (7)	0.0044 (7)	−0.0047 (7)
C7	0.0384 (9)	0.0370 (9)	0.0394 (9)	−0.0139 (7)	0.0063 (7)	−0.0058 (7)
C8	0.0359 (8)	0.0346 (8)	0.0340 (8)	−0.0112 (7)	0.0044 (7)	−0.0043 (7)
C9	0.0459 (10)	0.0403 (9)	0.0420 (9)	−0.0179 (8)	0.0111 (8)	−0.0083 (7)
C10	0.0540 (11)	0.0386 (9)	0.0459 (10)	−0.0221 (8)	0.0124 (8)	−0.0107 (8)
C11	0.0495 (10)	0.0340 (9)	0.0435 (10)	−0.0126 (8)	0.0118 (8)	−0.0075 (7)
C12	0.0390 (9)	0.0358 (8)	0.0335 (8)	−0.0138 (7)	0.0046 (7)	−0.0068 (7)
C13	0.0375 (8)	0.0347 (8)	0.0324 (8)	−0.0128 (7)	0.0042 (7)	−0.0061 (7)
C14	0.0412 (9)	0.0366 (9)	0.0341 (8)	−0.0140 (7)	0.0048 (7)	−0.0060 (7)
C15	0.0501 (10)	0.0369 (9)	0.0318 (8)	−0.0172 (8)	0.0051 (7)	−0.0079 (7)
C16	0.0546 (11)	0.0410 (10)	0.0474 (10)	−0.0159 (9)	0.0080 (8)	−0.0092 (8)
C17	0.0697 (13)	0.0384 (10)	0.0611 (12)	−0.0105 (10)	0.0032 (10)	−0.0101 (9)
C18	0.0920 (16)	0.0391 (10)	0.0557 (12)	−0.0284 (11)	−0.0012 (11)	−0.0016 (9)
C19	0.0783 (14)	0.0561 (12)	0.0498 (11)	−0.0386 (11)	0.0104 (10)	−0.0032 (9)
C20	0.0570 (11)	0.0467 (10)	0.0413 (10)	−0.0223 (9)	0.0104 (8)	−0.0075 (8)
C21	0.0427 (9)	0.0415 (9)	0.0376 (9)	−0.0163 (8)	0.0123 (7)	−0.0071 (7)
C22	0.0380 (9)	0.0380 (9)	0.0351 (9)	−0.0116 (7)	0.0053 (7)	−0.0081 (7)
C23	0.0385 (9)	0.0375 (9)	0.0413 (9)	−0.0156 (7)	0.0098 (7)	−0.0089 (7)
C24	0.0453 (10)	0.0474 (10)	0.0414 (10)	−0.0088 (8)	0.0056 (8)	−0.0087 (8)
C25	0.0536 (11)	0.0579 (12)	0.0438 (10)	−0.0153 (9)	0.0106 (9)	−0.0192 (9)
C26	0.0459 (10)	0.0398 (10)	0.0579 (11)	−0.0161 (8)	0.0174 (9)	−0.0149 (8)
C27	0.0452 (10)	0.0391 (10)	0.0522 (11)	−0.0094 (8)	0.0067 (8)	0.0004 (8)
C28	0.0490 (10)	0.0446 (10)	0.0387 (9)	−0.0144 (8)	0.0093 (8)	−0.0049 (8)

### Geometric parameters (Å, °)

**Table:**

Cl1—C3	1.7366 (17)	C12—C22	1.397 (2)
Cl2—C26	1.7367 (17)	C12—C13	1.405 (2)
N1—C14	1.337 (2)	C14—C21	1.389 (2)
N1—C13	1.342 (2)	C14—C15	1.487 (2)
C1—C2	1.378 (2)	C15—C20	1.387 (2)
C1—C6	1.395 (2)	C15—C16	1.389 (2)
C1—H1B	0.9300	C16—C17	1.380 (3)
C2—C3	1.368 (3)	C16—H16A	0.9300
C2—H2A	0.9300	C17—C18	1.376 (3)
C3—C4	1.373 (3)	C17—H17A	0.9300
C4—C5	1.375 (2)	C18—C19	1.373 (3)
C4—H4A	0.9300	C18—H18A	0.9300
C5—C6	1.393 (2)	C19—C20	1.383 (3)
C5—H5A	0.9300	C19—H19A	0.9300
C6—C7	1.465 (2)	C20—H20A	0.9300
C7—C8	1.343 (2)	C21—C22	1.386 (2)
C7—H7A	0.9300	C21—H21A	0.9300
C8—C13	1.489 (2)	C22—C23	1.487 (2)
C8—C9	1.513 (2)	C23—C28	1.382 (2)
C9—C10	1.518 (2)	C23—C24	1.387 (2)
C9—H9A	0.9700	C24—C25	1.382 (2)
C9—H9B	0.9700	C24—H24A	0.9300
C10—C11	1.517 (2)	C25—C26	1.372 (3)
C10—H10A	0.9700	C25—H25A	0.9300
C10—H10B	0.9700	C26—C27	1.371 (3)
C11—C12	1.505 (2)	C27—C28	1.382 (2)
C11—H11A	0.9700	C27—H27A	0.9300
C11—H11B	0.9700	C28—H28A	0.9300
			
C14—N1—C13	118.91 (14)	N1—C13—C8	116.56 (13)
C2—C1—C6	122.23 (17)	C12—C13—C8	120.52 (14)
C2—C1—H1B	118.9	N1—C14—C21	121.64 (15)
C6—C1—H1B	118.9	N1—C14—C15	116.46 (14)
C3—C2—C1	119.02 (17)	C21—C14—C15	121.88 (14)
C3—C2—H2A	120.5	C20—C15—C16	118.21 (16)
C1—C2—H2A	120.5	C20—C15—C14	121.62 (16)
C2—C3—C4	120.79 (16)	C16—C15—C14	120.16 (15)
C2—C3—Cl1	119.54 (14)	C17—C16—C15	120.80 (18)
C4—C3—Cl1	119.66 (15)	C17—C16—H16A	119.6
C3—C4—C5	119.74 (18)	C15—C16—H16A	119.6
C3—C4—H4A	120.1	C18—C17—C16	120.35 (19)
C5—C4—H4A	120.1	C18—C17—H17A	119.8
C4—C5—C6	121.59 (17)	C16—C17—H17A	119.8
C4—C5—H5A	119.2	C19—C18—C17	119.53 (18)
C6—C5—H5A	119.2	C19—C18—H18A	120.2
C5—C6—C1	116.61 (15)	C17—C18—H18A	120.2
C5—C6—C7	126.52 (15)	C18—C19—C20	120.41 (19)
C1—C6—C7	116.83 (15)	C18—C19—H19A	119.8
C8—C7—C6	131.97 (16)	C20—C19—H19A	119.8
C8—C7—H7A	114.0	C19—C20—C15	120.70 (19)
C6—C7—H7A	114.0	C19—C20—H20A	119.7
C7—C8—C13	117.97 (14)	C15—C20—H20A	119.7
C7—C8—C9	124.69 (14)	C22—C21—C14	120.16 (15)
C13—C8—C9	117.31 (13)	C22—C21—H21A	119.9
C8—C9—C10	113.80 (13)	C14—C21—H21A	119.9
C8—C9—H9A	108.8	C21—C22—C12	118.56 (15)
C10—C9—H9A	108.8	C21—C22—C23	119.49 (14)
C8—C9—H9B	108.8	C12—C22—C23	121.95 (14)
C10—C9—H9B	108.8	C28—C23—C24	118.10 (16)
H9A—C9—H9B	107.7	C28—C23—C22	121.24 (15)
C11—C10—C9	111.40 (14)	C24—C23—C22	120.66 (16)
C11—C10—H10A	109.3	C25—C24—C23	121.08 (17)
C9—C10—H10A	109.3	C25—C24—H24A	119.5
C11—C10—H10B	109.3	C23—C24—H24A	119.5
C9—C10—H10B	109.3	C26—C25—C24	119.17 (17)
H10A—C10—H10B	108.0	C26—C25—H25A	120.4
C12—C11—C10	108.64 (14)	C24—C25—H25A	120.4
C12—C11—H11A	110.0	C27—C26—C25	121.19 (17)
C10—C11—H11A	110.0	C27—C26—Cl2	118.83 (15)
C12—C11—H11B	110.0	C25—C26—Cl2	119.95 (15)
C10—C11—H11B	110.0	C26—C27—C28	119.02 (17)
H11A—C11—H11B	108.3	C26—C27—H27A	120.5
C22—C12—C13	117.77 (14)	C28—C27—H27A	120.5
C22—C12—C11	123.25 (14)	C23—C28—C27	121.37 (16)
C13—C12—C11	118.81 (14)	C23—C28—H28A	119.3
N1—C13—C12	122.92 (14)	C27—C28—H28A	119.3

### Hydrogen-bond geometry (Å, °)

**Table:**

*D*—H···*A*	*D*—H	H···*A*	*D*···*A*	*D*—H···*A*
C7—H7A···N1	0.93	2.34	2.760 (2)	107
C20—H20A···Cl1^i^	0.93	2.80	3.476 (2)	130

Symmetry codes: (i) *x*−1, *y*, *z*+1.
